# Unmet Needs in Dementia Care: The Predictive Role of Caregiver Challenges

**DOI:** 10.1002/gps.70164

**Published:** 2025-10-16

**Authors:** Laiss Bertola, Fabiana da Mata, Ari Alex Ramos, Carolina Godoy, Fernanda Menezes de Faria, Rosa Lucchetta, Haliton Alves de Oliveira Junior, Cleusa Pinheiro Ferri

**Affiliations:** ^1^ Hospital Alemão Oswaldo Cruz Research Unit Responsabilidade Social São Paulo Brazil; ^2^ Departmet of Psychiatry Medical School Universidade Federal de São Paulo São Paulo Brazil; ^3^ Department of Gerontology Universidade Federal de São Carlos São Paulo Brazil

**Keywords:** caregiver, dementia, low and middle‐income countries, unmet care needs

## Abstract

**Background and Objectives:**

In low‐ and middle‐income countries (LMICs), family caregivers provide most of the support for people with dementia (PWD), yet little is known about how caregiver needs relate to unmet needs of PWD ^–^ especially in Latin America. This study explored that relationship in Brazilian caregiver‐PWD dyads.

**Methods:**

140 Brazilian dyads underwent in‐home interviews, with assessments including a comprehensive needs of care instrument, caregiver burden, mental health, and caregiving experience, as well as PWD symptoms and demographics.

**Results:**

All dyads had at least one unmet need. A combined model incorporating predictors from both PWD and caregivers indicated that PLWD having lower education, being male, and exhibiting more neuropsychiatric symptoms are associated with more unmet needs. Additionally, caregiver‐related factors such as being male, having fewer years of caregiving experience, and having more unmet needs of their own were also linked to greater unmet needs in PWD.

**Discussion and Implications:**

Caregiver well‐being significantly affects the quality of dementia care. Caregivers' unmet needs likely reflect both their personal limitations and broader care challenges. Addressing both caregiver and PWD needs through targeted support strategies is essential for improving dementia care in LMICs, and especially in culturally and economically diverse settings.

## Key Points

- All Caregiver‐People with Dementia (PWD) dyads had unmet needs, demonstrating substantial deficiencies in the dementia care framework.
- The total caregiver unmet needs is a significant and unique predictor of a higher number of unmet needs in PWD, suggesting that the general well‐being and limitations of the caregiver directly dictate care quality.
- PWD with lower education, male gender, and more neuropsychiatric symptoms are significantly associated with a greater number of unmet needs.
- Caregiver characteristics predicting greater unmet needs in PWD include the caregiver being male and having fewer years of caregiving experience.
- Targeted support strategies must adopt dyadic and integrated approaches, prioritizing caregiver support and education to address the complex interplay between PWD attributes and caregiver unmet needs for effective dementia care.

## 1 Introduction

People with dementia (PWD) often experience numerous unmet needs, including difficulties with daily activities, social engagement, emotional well‐being, and access to healthcare [1, 2]. When these needs remain unaddressed, they can lead to negative outcomes such as increased behavioral and psychological symptoms of dementia (BPSD), reduced quality of life, higher levels of distress, and even accelerated cognitive decline [3, 4]. Furthermore, unmet needs may contribute to a higher likelihood of institutionalization, placing additional strain on both healthcare systems and family caregivers [5, 6].

In low‐ and middle‐income countries (LMICs), where formal support systems for dementia care are often underdeveloped, family caregivers typically bear the primary responsibility for providing care. Absence of sufficient institutional, financial, and psychological support increases the risk of caregiver burden [7, 8]. Moreover, prolonged stress can result in significant psychiatric symptoms including anxiety, depression, and burnout. Hence, without appropriate interventions, caregivers may struggle to maintain their own well‐being while providing necessary care for PWD.

The mental health status of caregivers plays a crucial role in the quality of care they provide. When caregivers experience high levels of stress or psychological distress, they may find it difficult to effectively address the needs of PWD [9, 10]. Emotional exhaustion can lead to reduced patience, less engagement, and an overall decline in quality of support. Conversely, caregivers with adequate mental health resources and coping strategies are more likely to provide attentive and compassionate care, ultimately benefiting both themselves and the individuals they support.

Unmet needs are not limited to PWD; caregivers also have their own set of needs that, if neglected, can exacerbate the caregiving burden [11, 12]. Care needs is a broad and multidimensional term that encompasses the perception that specific spheres of an individual's life may be affected and impaired by health conditions ^–^ not only mental health, thereby negatively impacting their well‐being and other outcomes [13]. Addressing the dyadic nature of dementia care, in which the needs of both PWD and their caregivers are considered, is essential for a holistic approach to improve outcomes. By analyzing this dynamic relationship, interventions can be tailored to ensure that both parties receive appropriate support and foster a more sustainable caregiving environment.

Despite the growing recognition of care‐related challenges in dementia, understanding the interplay between caregiver mental health, their unmet needs and the unmet needs of PWD, particularly in Latin America, is still scarce [14]. Specifically, there is a gap in the literature in analyzing the impact of caregivers' unmet needs beyond their mental health and burden. This manuscript aims to explore this relationship, emphasizing the importance of addressing both caregivers and PWD needs to enhance overall well‐being and care outcomes.

## 2 Methods

### 2.1 Study Design, Setting and Participants

ReNaDe Project (National Report on Dementia in Brazil) is a quantitative cross‐sectional study (ethical approval CAAE n°. 58,125,222.6.0000.0070). The project enrolled 140 dyads of people with dementia and their primary caregivers who had a previous diagnosis of dementia and used mainly the Brazilian Unified Health System (SUS) for care. The dyads were selected from 17 cities of different population sizes and from all five geographic regions of Brazil, considering small‐sized municipalities (with a population of up to 50,000), medium‐sized (between 51,000 and 99,999), and large‐sized (100,000 or more). Individuals living with dementia had to be at least 60 years old and have an ICD‐10 diagnosis of dementia. The included caregivers had to be the main caregivers of PWD (regardless of the familial relationship), cognitively intact, and able to answer the interview. In the context of the ReNaDe Project, caregivers were defined as persons who provided assistance to a person living with dementia in daily life activities, who were not formally employed as caregivers nor were paid for caregiving services (i.e., family or friends).

The dyads underwent in‐home interviews conducted by the clinical research team between 2022 and 2023. The research team consisted of two neuropsychologists and one physical therapist, all with doctoral‐level gerontology training. Each dyad was interviewed by one professional. Household interviews lasted an average of 90 min, during which the team collected data on sociodemographic characteristics, general health conditions, and administered several standardized instruments (described below). Most questions were addressed to the caregiver.

### 2.2 Needs of Care—Johns Hopkins Dementia Care Needs Assessment—Brazilian Version (JHDCNA‐Br 2.0)

To assess the need for care, we used the translated and adapted Brazilian Portuguese version [15] of the Johns Hopkins Dementia Care Needs Assessment (JHDCNA, 2.0) [5, 6]. The JHDCNA‐Br 2.0 comprises 62 items divided into seven domains related to people with dementia needs (44 items) and six domains related to caregiver needs (18 items). A trained health clinician must document the met or unmet needs of PWD in the following seven domains: (1) Cognitive Symptom Management (3 items), (2) Neuropsychiatric Symptom Management (5 items), (3) General Health Care (10 items), (4) Home and Personal Safety (10 items), (5) Daily Activities (7 items), (6) Legal Concerns/Advanced Care Planning (4 items), and (7) Health Care Financing (4 items). For the caregiver, the domains were as follows: (1) Caregiver Education (8 items), (2) Mental Health Care (3 items), (3) General Health Care (2 items), (4) Informal Support (1 item), (5) Daily Living (3 items), and (6) Legal Concerns (1 item).

Health clinicians only completed the JHDCNA‐Br 2.0 after conducting interviews with the caregivers and people with dementia, a visual evaluation of the PWD at home, and considering individuals' perspectives on their needs. JHDCNA does not contain reverse‐coded items. Each JHDCNA item is assessed as being needed and, if needed, whether the need is fully met, partially met, or unmet, scored as 0‐no need, 1‐fully met need, 2‐partially met need and 3‐unmet need. Item scoring can also be transformed into a dichotomic 0‐no need or fully met need and 1‐partially met or unmet need. In this study, this dichotomous scoring was used.

### 2.3 Variables and Other Measures

Regarding information about the PWD, in addition to the JHDCNA‐Br 2.0 (described previously), the team collected data on sociodemographic characteristics, general health conditions, and other instruments, as listed below.

– Age (years), education (years), race (white or black/brown), per capita income.
– 17 medical conditions were summed up to calculate the total number of diagnosed diseases: diabetes, high cholesterol, hypertension, heart attack, angina, heart failure, stroke, asthma, emphysema/bronchitis/chronic obstructive pulmonary disease (COPD), arthritis/rheumatism, osteoporosis, chronic back problems, depression, cancer, chronic kidney failure, Parkinson's disease, and epilepsy/seizures.
– The Neuropsychiatric Inventory (NPI) assesses the presence and intensity of 12 neuropsychiatric symptoms [16]. Each symptom score is calculated as the product of frequency and severity, and the final score is obtained by summing of all the 12 symptom scores.
– Katz and Pfeffer scales are indices of basic and instrumental activities of daily living, respectively [17]. Each item is scored as independent, partially dependent, or dependent and the final score is the sum of all items within each index.
– Clinical Dementia Rating to classify dementia stage [18] is commonly performed: 0, normal; 0.5, questionable dementia; 1, initial dementia; 2, moderate dementia; and 3, severe dementia.

In addition, for caregivers sociodemographic variables (age, sex, education, and relationship with the PWD), caring‐related questions (existence of a backup caregiver, number of additional caregivers, years as the primary caregiver, daily time spent providing support for basic and instrumental activities of daily living) and scales were administered. These included the Zarit Burden Interview to assess subjective care‐related burden [19] and the Self‐Report Questionnaire (SRQ20) to measure common psychiatric symptoms of depression and anxiety [20].

### 2.4 Data Analysis

Descriptive data analyses were performed using the mean, standard deviation, or frequency according to variable characteristics. To elucidate the variables associated with a greater number of unmet needs in PWD (outcome), we conducted regression analyses following a three‐step approach: first, examining only the characteristics of the PWD; second, analyzing only the characteristics of the caregivers; and third, investigating a combination of characteristics from both the PWD and their caregivers.

The first set of analyses was divided into two models. In Model 1, only the basic sociodemographic characteristics of PWD (age, education, gender, ethnicity/race, and per capita income) were included. In Model 2, the variables from Model 1 were supplemented with clinical characteristics (CDR and multimorbidity), degree of dependency in basic and instrumental activities of daily living, and neuropsychiatric symptoms.

The second set of analyses, considering only caregiver characteristics as predictors of unmet care needs for PWD, was divided into three models. In Model 1, basic sociodemographic characteristics were considered along with mental health symptoms and caregiver burden. In Model 2, the same variables from Model 1 were included but supplemented with information about the caregiving context (presence of a backup caregiver, number of additional caregivers, time spent assisting with basic and instrumental activities of daily living, and the number of years the caregiver has been providing care). In Model 3, we added the total number of unmet needs of the caregivers.

Finally, in one unique model, the third set of regression analyses considered previous predictors that were significant in the first and second sets of analysis.

## 3 Results

PWD had a mean age of 81.3 years, a mean education of 3.7 years, and 69% were women (Table 1). Caregivers had a mean age of 57.8 years, with a mean education of 8 years, 86% were women and 57% were an adult child of the PWD (Table 2).

**TABLE 1:** People living with dementia characteristics.

	Overall (*N* = 140)
Age ‐ mean (SD)	81.3 (7.9)
Education (years) – Mean (SD)	3.7 (3.7)
Sex (woman) – *N* (%)	97 (69.3%)
Race (white) – *N* (%)	85 (60.7%)
Per capita income (minimum wage) – Mean (SD)	1.3 (1.0)
CDR – *N* (%)	
1 – Mild	31 (22.1%)
2 – Moderate	53 (37.9%)
3 – Advanced	56 (40.0%)
Neuropsychiatric symptoms – mean (SD)	22.2 (15.8)
Multimorbidity – Mean (SD)	3.6 (2.0)
ADLs	6.3 (4.9)
IADLs	26.4 (6.5)

*Note:* One minimum wage, at data collection, was equivalent to 1200 Brazilian reais, equivalent to 230 US dollars.

Abbreviations: ADL: basic activities of daily living; CDR ^–^ Clinical Dementia Rating: 1 – mild, 2 – moderate, 3 – advanced; IADL: instrumental activities of daily living; SD: standard deviation.

**TABLE 2:** Caregivers' characteristics.

	Overall (*N* = 140)
Age – mean (SD)	57.8 (12.6)
Sex (woman) – *N* (%)	121 (86.4%)
Race (white) – *N* (%)	78 (55.7%)
Education (Years) – Mean (SD)	8.6 (4.8)
Time as a caregiver (Years) – Mean (SD)	5.1 (5.0)
Relationship with the PWD	
Spouse	36 (25.7%)
Child	80 (57.1%)
Backup caregiver (No) – *N* (%)	42 (30.0%)
Number of additional caregivers – Mean (SD)	1.8 (1.6)
Burden – Mean (SD)	31.3 (15.2)
Common mental symptoms (depression and anxiety) – Mean (SD)	6.7 (4.7)
Time spent in ADLs (hours/day) – Mean (SD)	3.1 (2.7)
Time spent in IADLs (hours/day) – Mean (SD)	4.5 (2.3)

*Note:* One minimum wage, at data collection, was equivalent to 1200 Brazilian reais, equivalent to 230 US dollars.

Abbreviations: ADL: basic activities of daily living; IADL: instrumental activities of daily living; PWD: people with dementia; SD: standard deviation.

PWD had a mean of 18.1 (SD = 6.9) unmet needs (minimum = 2 and maximum = 36), while caregivers had a mean of 11.4 (SD = 3.9) unmet needs (minimum = 2 and maximum = 18), which revealed both caregiver and PWD had unmet needs and no dyad had all needs met.

Regression analysis showed that in the first set ‐ considering only PWD characteristics ‐ the predictors of more unmet needs were lower education (*β* = −0.62, *p* = 0.002), being male (*β* = −3.06, *p* = 0.044), and exhibiting more neuropsychiatric symptoms (*β* = 0.12, *p* = 0.004) (Table 3).

**TABLE 3:** People with dementia unmet needs – people with dementia predictors (*N* = 140).

	Model 1			Model 2		
Predictors	Coefficient (*β*) (CI 95%)	Standard error (SE)	*p*‐value	Coefficient (β) (CI 95%)	Standard error (SE)	*p*‐value
Constant	**27.80 (13.84**–**41.77)**	**7.04**	**< 0.001**	**23.55 (7.55**–**39.54)**	**8.05**	**0.004**
Age	−0.06 (−0.23–0.11)	0.09	0.500	0.01 (−0.18–0.19)	0.09	0.940
Education (years)	**−0.63 (−1.02**–**−0.25)**	**0.19**	**0.002**	**−0.62 (**−**1.00**–**−0.23)**	**0.19**	**0.002**
Sex (woman)	**−3.65 (−6.46**–−**0.84)**	**1.42**	**0.012**	**−3.06 (**−**6.04**–−**0.08)**	**1.50**	**0.044**
Race (black/Brown)	**2.63 (0.08**–**5.17)**	**1.28**	**0.043**	2.50 (−0.12–5.11)	1.32	0.061
Per capita income (salaries)	−0.86 (−2.24–0.53)	0.70	0.223	−0.65 (−2.02–0.73)	0.69	0.352
CDR 2				0.71 (−3.12–4.53)	1.93	0.715
CDR 3				−2.11 (−7.47–3.24)	2.70	0.436
Number of diseases (multimorbidity)				−0.49 (−1.14–0.16)	0.33	0.138
ADLs				0.10 (−0.37–0.56)	0.23	0.682
IADLs				−0.10 (−0.36–0.17)	0.13	0.464
Neuropsychiatric symptoms				**0.12 (0.04**–**0.20)**	**0.04**	**0.004**
*R* ^2^/*R* ^2^ adjusted	0.250/0.213			0.378/0.302		

*Note:* At the time of data collection, one salary corresponded to 1200 Brazilian reais, approximately 230 US dollars.

Abbreviations: ADL: basic activities of daily living; CDR: Clinical Dementia Rating: 1 – mild (reference), 2 – moderate, 3 – advanced; IADL: instrumental activities of daily living.

Regression analyses from the second set, which considered only caregiver characteristics as predictors (Table 4), revealed that in Model 1, younger age, male gender, lower education, and higher levels of caregiver burden were associated with more PWD unmet needs. In Model 2, lower education and higher burden remained predictors, along with fewer years of caregiving experience. In Model 3, male gender (*β* = −3.02, *p* = 0.036), fewer years of caregiving experience (*β* = −0.21, *p* = 0.035), and more self‐reported unmet needs (*β* = 1.18, *p* < 0.001) were identified as predictors of a higher number of unmet needs for PWD.

**TABLE 4:** People with dementia unmet needs – caregivers predictors (*N* = 140).

	Model 1			Model 2			Model 3		
Predictors	Coefficient (*β*) (CI 95%)	Standard error (SE)	*p*‐value	Coefficient (*β*) (CI 95%)	Standard error (SE)	*p*‐value	Coefficient (*β*) (CI 95%)	Standard error (SE)	*p*‐value
Constant	**25.07 (15.90**–**34.24)**	**4.64**	**< 0.001**	**26.94 (16.98** **–** **36.90)**	**5.03**	**< 0.001**	**12.35 (3.03**–**21.66)**	**4.70**	**0.010**
Age (caregiver)	**−0.11 (−0.22**–**−0.00)**	**0.06**	**0.045**	−0.09 (−0.20–0.03)	0.06	0.145	−0.05 (−0.14–0.05)	0.05	0.367
Sex (caregiver) (woman)	**−3.35 (**−**6.60**–−**0.11)**	**1.64**	**0.043**	−2.70 (−6.05–0.65)	1.69	0.114	**−3.02 (−5.84**–**−0.19)**	**1.43**	**0.036**
Education (caregiver) (years)	**−0.30 (−0.56**–**−0.04)**	**0.13**	**0.023**	**−0.28 (−0.54**–**−0.01)**	**0.13**	**0.042**	−0.10 (−0.33–0.12)	0.12	0.369
Relationship ‐ Son/Daughter	0.01 (−3.24–3.27)	1.65	0.994	1.07 (−2.43–4.58)	1.77	0.545	1.16 (−1.78–4.11)	1.49	0.436
Relationship ‐ other	0.70 (−3.33–4.73)	2.04	0.732	1.27 (−3.07–5.61)	2.19	0.562	1.52 (−2.14–5.17)	1.85	0.413
Burden	**0.16 (0.06**–**0.25)**	**0.05**	**0.002**	**0.15 (0.05**–**0.26)**	**0.05**	**0.005**	0.08 (−0.01–0.17)	0.05	0.067
Common mental symptoms (depression and anxiety)	0.00 (−0.31–0.31)	0.16	0.998	0.05 (−0.27–0.37)	0.16	0.758	−0.28 (−0.57–0.00)	0.14	0.052
Backup caregiver				−1.78 (−4.55–0.99)	1.40	0.205	0.48 (−1.93–2.90)	1.22	0.691
Additional caregivers				−0.07 (−0.82–0.69)	0.38	0.863	−0.06 (−0.69–0.58)	0.32	0.863
Time spent on ADLs (hours/day)				−0.12 (−0.55–0.32)	0.22	0.592	−0.20 (−0.56–0.17)	0.18	0.283
Time spent on IADLs (hours/day)				−0.34 (−0.86–0.18)	0.26	0.203	−0.40 (−0.84–0.04)	0.22	0.077
Time as a caregiver (years)				**−0.36 (−0.59**–**−0.12)**	**0.12**	**0.003**	**−0.21 (−0.41**–**−0.01)**	**0.10**	**0.035**
Unmet needs of the caregiver							**1.18 (0.85**–**1.51)**	**0.17**	**< 0.001**
*R* ^2^/*R* ^2^ adjusted	0.183/0.140			0.250/0.176			0.473/0.416		

Abbreviations: ADL: basic activities of daily living; IADL: instrumental activities of daily living.

Finally, the third set included predictors of unmet needs in PWD, incorporating both characteristics of PWD (education, sex, and neuropsychiatric symptoms) and of the caregiver (sex, education, burden, mental health symptoms, number of years the caregiver has been providing care, and unmet needs of the caregiver) (Table 5). In this combined model, factors associated with a higher number of unmet needs for PWD included lower education (*β* = −0.52, *p* < 0.001), male gender (*β* = −2.13, *p* = 0.043), and more neuropsychiatric symptoms (*β* = 0.09, *p* = 0.005), as well as caregiver characteristics such as male gender (*β* = −3.64, *p* = 0.006), having fewer years of caregiving experience (*β* = −0.20, *p* = 0.031), and more self‐reported unmet needs (*β* = 1.02, *p* < 0.001).

**TABLE 5:** People with dementia unmet needs – combined predictors (*N* = 140).

	Model		
Predictors	coefficient (*β*) (CI 95%)	Standard error (SE)	*p*‐value
Constant	**12.35 (7.63**–**17.06)**	**2.38**	**< 0.001**
Education (years)	**−0.52 (‐0.78**–−**0.26)**	**0.13**	**< 0.001**
Sex (woman)	**−2.13 (−4.20**–**−0.06)**	**1.04**	**0.043**
Neuropsychiatric symptoms	**0.09 (0.03**–**0.15)**	**0.03**	**0.005**
Sex (caregiver) (woman)	**−3.64 (−6.21**–**−1.07)**	**1.30**	**0.006**
Education (caregiver) (years)	0.11 (−0.10–0.32)	0.10	0.291
Burden	0.00 (−0.08–0.09)	0.04	0.938
Common mental symptoms (depression and anxiety)	−0.20 (−0.47–0.07)	0.14	0.145
Time as a caregiver (years)	**−0.20 (−0.38**–**−0.02)**	**0.09**	**0.031**
Unmet needs of the caregiver	**1.02 (0.72–1.33)**	**0.15**	**< 0.001**
*R* ^2^/*R* ^2^ adjusted	0.535/0.501		

## 4 Discussion

Our findings indicate that all dyads had at least two unmet needs per individual, with the total unmet needs constituting approximately half of the total needs assessed. This highlights gaps in dementia care and the necessity for more structured and holistic interventions to address these deficiencies. Other studies have reported similar results, highlighting that unmet needs in dementia are a rampant reality [5, 21].

Notably, caregivers' unmet needs emerged as a significant predictor of the need for PWD, instead of burden, the most well‐known caregiver predictor. This finding suggests that general caregiver well‐being directly influences the quality of care they provide. Caregiver needs likely reflect not only their mental health and burden status but also their overall personal and situational capacity to provide care, including access to resources for self‐care and their physical, mental, and social quality of life [22, 23, 24]. A caregiver struggling with their own unmet needs may be less equipped to adequately meet the demands of a person with dementia, exacerbating challenges in care provision.

Most previous results available in literature highlight that caregiver burden is associated with more unmet needs for people with dementia [9, 14]. However, our results makes an unique contribution to dementia research in Latin America by underlying that the general unmet needs of caregivers might be a better predictor for care and thus should be taken into account [12]. In this regard, burden might be a product not only to the role of carer itself, but also to the lack of time and availability to a broader selfcare during this role.

Certain characteristics of PWD are also associated with a higher number of unmet needs. Specifically, lower education level, male gender, and increased neuropsychiatric symptoms were significant predictors. Individuals with lower education may have less access to health literacy and resources, making it more challenging to navigate care systems, understand their own needs, and advocate for appropriate services [1, 25, 26].

Additionally, male PWD may struggle to express their needs effectively due to culturally expected gender roles and may also experience different social expectations or support dynamics that result in more unmet needs. e.g., in primary care, men usually seek less medical advice than women [27, 28].

More severe neuropsychiatric symptoms predicting more unmet needs have been extensively reported [5, 29]. Neuropsychiatric symptoms likely contribute to increased caregiving demands, making it harder for caregivers to fully address all needs [1]. In addition, managing challenging behaviors requires more knowledge and techniques, which the familial caregiver often does not have. In our sample, caregiver education about dementia management was a frequent unmet need [15].

Regarding caregivers, male gender and fewer years of caregiving experience were associated with greater unmet needs. Male caregivers may have different social support structures [30] or levels of preparedness compared to female caregivers due to culturally expected gender roles. Male carers might also approach help‐seeking differently than female carers, focusing more on addressing functional tasks and abstaining from addressing behavioral and emotional tasks [31], which might contribute to increased unmet needs. Additionally, considering that caregivers' education about dementia and caring techniques is a major unmet need, those with less experience may have less time to learn effective caregiving techniques with daily practice, but also be less familiar with available resources and coping strategies, further contributing to care deficits.

In this sample, we were unable to ascertain the dementia type due to a lack of access to medical records, and most families were unaware of the specific type of dementia, thereby introducing a limitation to the present study. Since dementia type may influence the extent and nature of caregiving needs, and it has already been associated with caregiver burden, the lack of this information might limit the comprehension of the predictors of unmet needs [32].

Despite this limitation, our findings underscore the importance of dyadic approaches in dementia care. Addressing both caregiver and PWD needs in an integrated manner is crucial for improving the overall outcomes. These findings can guide the development of training for primary care professionals, focusing on the importance of an integrated approach to address the needs of caregivers and PWD. Future interventions should prioritize caregiver support and education, particularly for those with less experience and limited access to resources. Strengthening support networks and mental health services for caregivers may in turn lead to improved care and reduced unmet needs for PWD. Ultimately, targeted strategies must account for the complex interplay between caregiver characteristics, PWD attributes, and the broader care environment to ensure a sustainable and effective dementia‐care framework.

## Ethics Statement

ReNaDe Project ‐ National Report on Dementia in Brazil ‐ Ethical approvement: CAAE n°. (58,125,222.6.0000.0070).

## Conflicts of Interest

The authors declare no conflicts of interest.

## Data Availability

The data that support the findings of this study are available on request from the corresponding author. The data are not publicly available due to privacy or ethical restrictions.
